# Severe Necrotizing Pneumonia Caused by Panton‑Valentine Leukocidin‑Producing Staphylococcus aureus in a Previously Healthy Adult

**DOI:** 10.7759/cureus.94097

**Published:** 2025-10-08

**Authors:** Prabin Paudyal, Pallavi Bedi

**Affiliations:** 1 Internal Medicine, University Hospital Hairmyres, NHS Lanarkshire, Glasgow, GBR; 2 Respiratory Medicine, University Hospital Hairmyres, NHS Lanarkshire, Glasgow, GBR

**Keywords:** necrotizing pneumonia, panton‑valentine leukocidin, respiratory failure, staphylococcus aureus, toxin suppression therapy

## Abstract

A previously healthy adult male presented with rapidly progressive pneumonia and systemic features of infection shortly after returning from travel abroad. Despite broad‑spectrum antimicrobial therapy and supportive measures, he deteriorated into respiratory failure requiring ICU admission and mechanical ventilation. Imaging revealed cavitating lung nodules and necrosis. Panton-Valentine leukocidin (PVL)‑positive Staphylococcus aureus (PVL‑SA) was confirmed by PCR. This case highlights the importance of early recognition and targeted management of PVL‑SA, a virulent toxin‑producing strain associated with severe necrotizing pneumonia and systemic complications, especially in young, otherwise healthy individuals.

## Introduction

Panton-Valentine leukocidin (PVL) is a bicomponent pore-forming cytotoxin encoded by the lukF-PV and lukS-PV genes, found in a minority of Staphylococcus aureus (S. aureus) strains [1,2]. This toxin has been implicated in severe clinical manifestations, particularly necrotizing pneumonia, which is often community-acquired and characterized by rapid progression and significant morbidity and mortality in affected patients, especially without timely intervention [2]. Clinical cases show that PVL-producing S. aureus can cause a spectrum of diseases ranging from mild skin and soft tissue infections to devastating necrotizing pneumonia, primarily affecting young, otherwise healthy individuals [3-6]. 

Patients experiencing necrotizing pneumonia often present first with flu-like symptoms or localized lesions that quickly escalate into life-threatening respiratory conditions, sometimes requiring mechanical ventilation due to respiratory failure [6,7]. The rapid deterioration and high mortality rates associated with this condition underline the importance of early recognition and immediate administration of appropriate antimicrobial therapies [6]. The pathological consequences of PVL are linked to its ability to induce leukocyte destruction and severe tissue necrosis [1]. Research indicates that PVL not only facilitates an inflammatory response but also contributes to significant lung damage, exacerbating the clinical picture in cases of pneumonia [5,8].

## Case presentation

A previously healthy adult male presented with shortness of breath, central non-radiating chest pain, fever (38.3°C), headache, vomiting, and myalgia. He had recently returned from Gran Canaria and reported a three-day history of a lesion on the back of his head, reminiscent of a previous episode of S. aureus sepsis 6-7 years earlier, which had required IV antibiotics and prolonged outpatient therapy.

**Figure 1:**
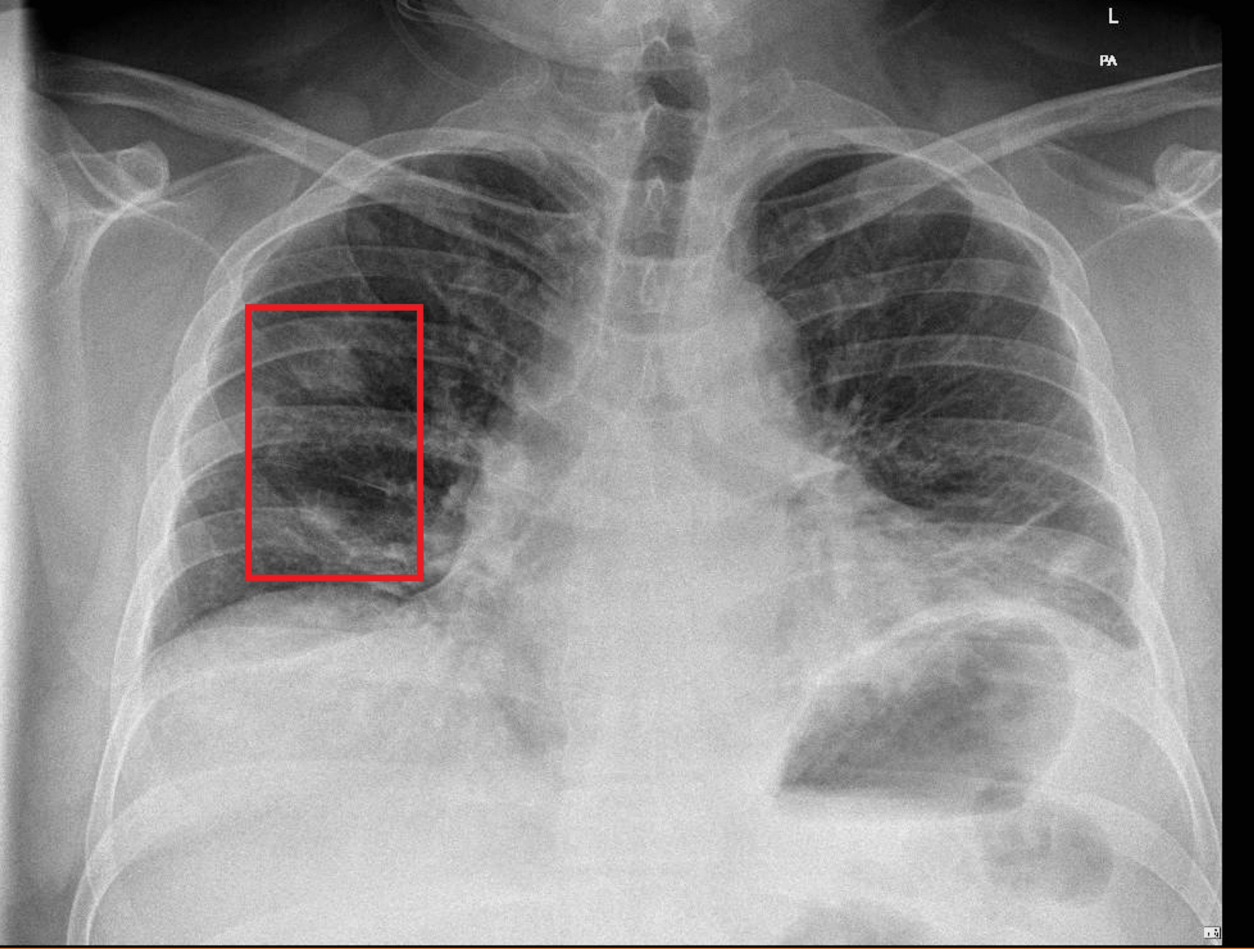
Chest X-ray on admission showing right middle zone opacity

On presentation, he was hypoxic with oxygen saturations of 92% (reference range: > 94%) on room air and showed signs of acute kidney injury (Cr 163; reference range: 60-120 umol/L, eGFR 41; reference range: >59 ml/min/1.73m^2^). Inflammatory markers were significantly elevated (CRP 343; reference range: < 6, WBC 17.5; reference range: 4.0 - 11.3 x10^9^/L), and a chest X-ray revealed an opacity in the right mid-zone (Figure 1). He was started on IV amoxicillin and tinzaparin in the Emergency Department (ED). The provisional diagnoses included lower respiratory tract infection or pulmonary embolism (PE).

**Figure 2:**
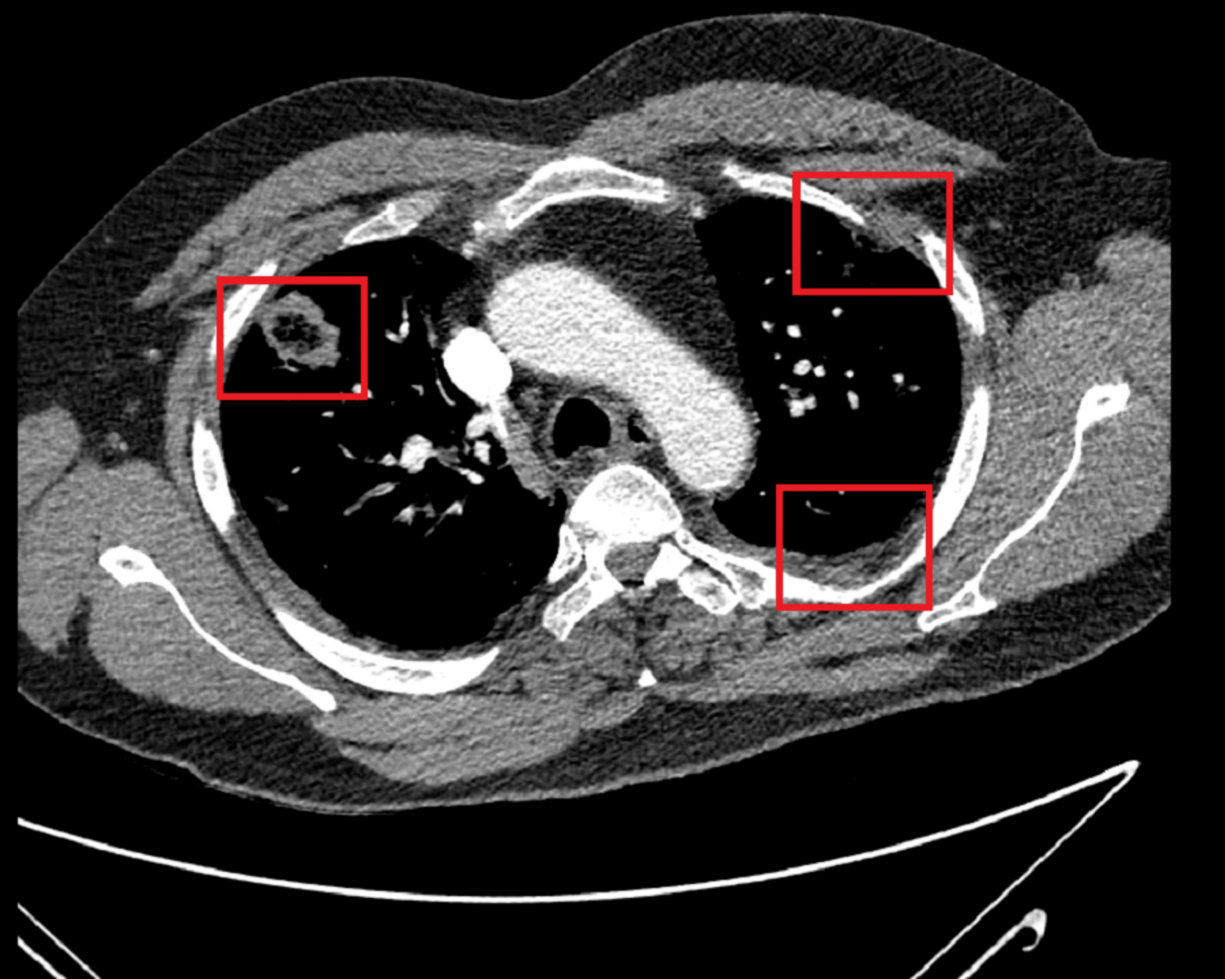
CT scan of the chest showing bilateral necrotizing pneumonia predominantly in the middle lung zones, with associated left-sided pleural effusion

By the following day, therefore, IV amoxicillin was escalated with addition of IV metronidazole and IV gentamicin. A CT pulmonary angiogram ruled out PE but showed bilateral pulmonary nodules with reverse halo signs and a small left-sided consolidation, concerning for a fungal or granulomatous disease (Figure 2). No intra-abdominal source of infection was identified on CT abdomen-pelvis.

**Figure 3:**
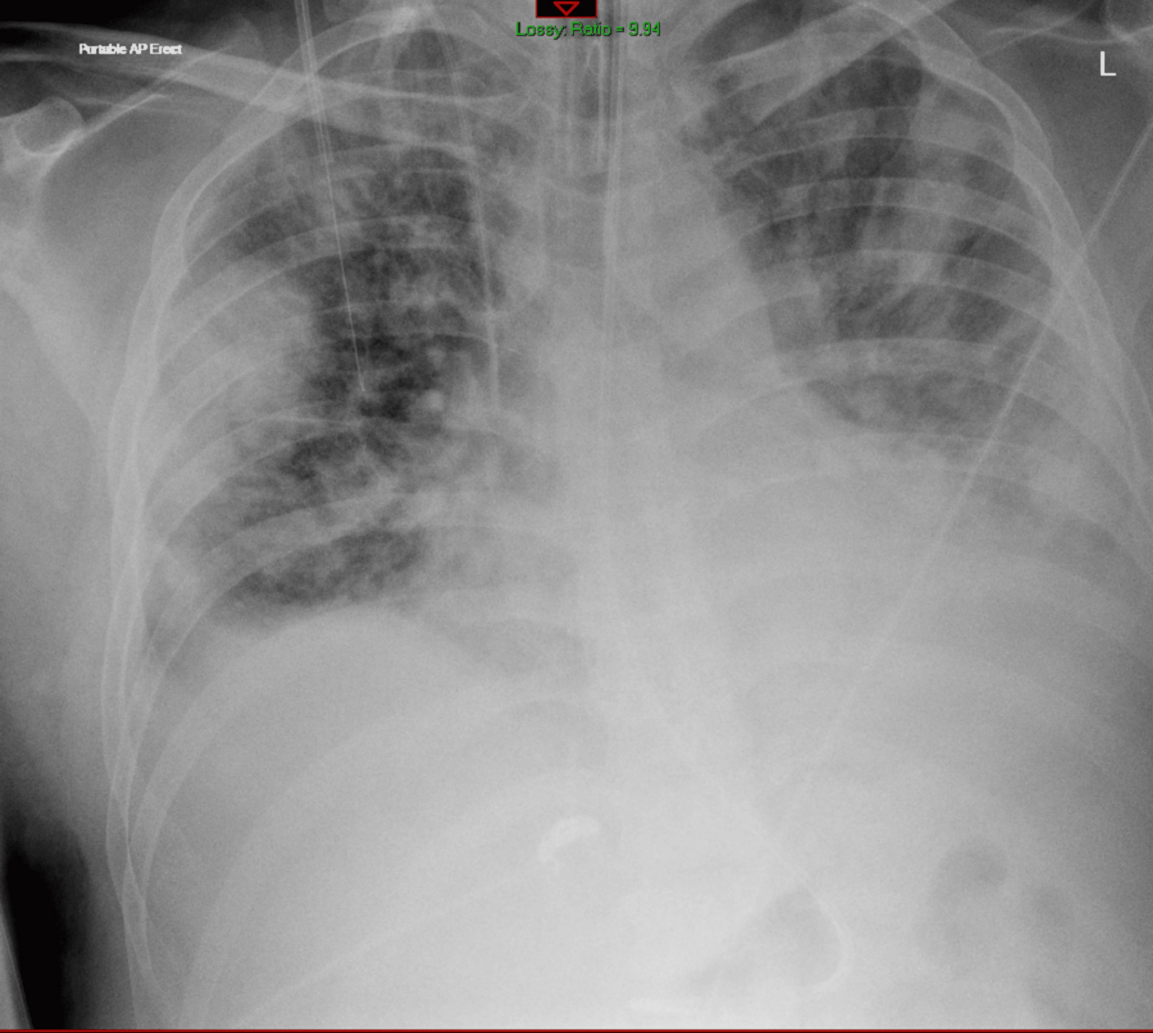
CXR showing progression of pneumonia in bilateral lungs

**Figure 4:**
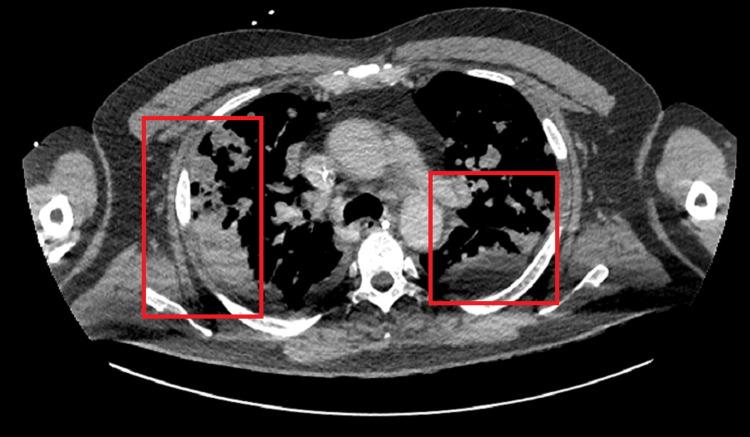
CT scan showing progression of bilateral necrotizing pneumonia in the middle lung zones, with increasing left-sided pleural effusion

**Figure 5:**
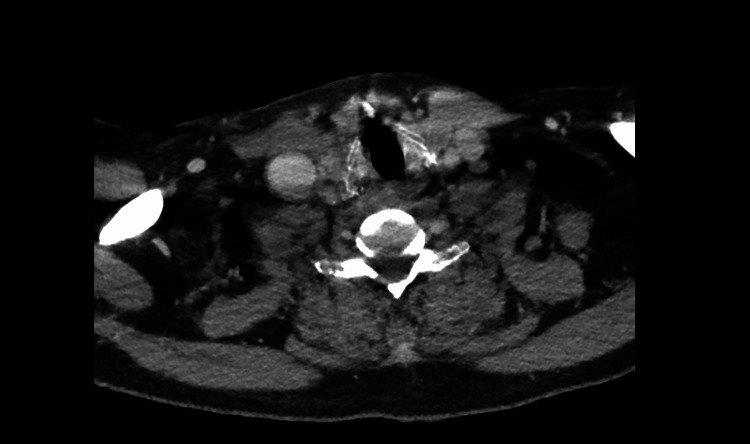
CT scan of the neck showing soft tissue edema surrounding the sternocleidomastoid muscle

Despite escalation of antibiotics, his condition deteriorated. He developed spiking fevers, gram-positive cocci were isolated from blood cultures, his AKI worsened, and CRP rose to 610. He was transferred to the Medical High Dependency Unit (MHDU) and started on meropenem and linezolid. By Day 3 of admission, he required high-flow nasal oxygen (60% FiO₂ at 60L/min) and exhibited signs of necrotizing pneumonia, with associated scalp abscesses and neck edema. Interval CXR (Figure 3) and CT chest (Figure 4) showed extensive perihilar shadowing and progression of pulmonary nodules with central necrosis. New neck lesions developed (Figure 5), prompting review by ENT and respiratory consultants, who requested HIV, galactomannan and Aspergillus IgE/IgG testing, along with a vasculitis screen, all of which tested negative. Caspofungin was added to his antimicrobial regimen, and an echocardiogram ruled out infective endocarditis. 

Due to his deteriorating respiratory status and signs of systemic infection, he was intubated and transferred to the Intensive Care Unit (ICU), where a central line and a nasogastric (NG) tube were placed on the third day of admission. His ongoing clinical deterioration and blood cultures suggesting gram-positive cocci raised suspicion of PVL-positive S. aureus, prompting a switch in antibiotics to linezolid, clindamycin, and rifampicin, with continuation of meropenem and caspofungin. Clarithromycin was stopped following a negative Legionella urinary antigen on the fourth day of admission. Over the following days, he remained hemodynamically stable without vasopressor support but developed increased respiratory secretions and agitation, managed with physiotherapy and a clonidine infusion. Candida was isolated from endotracheal secretions. TB cultures remained negative.

On ICU Day 5, persistent hypoxia and suspected pulmonary edema led to the initiation of IV furosemide. His antimicrobial therapy was adjusted: meropenem, linezolid, and clindamycin were discontinued, while flucloxacillin, rifampicin, and caspofungin were continued. Piperacillin/tazobactam was commenced at this stage. A significant drop in hemoglobin (Hb 60) necessitated PRBC transfusion, and a tracheostomy was planned due to anticipated prolonged ventilation. PVL-positive Staph aureus was confirmed by PCR on Day 10 of illness. Following Infectious Diseases consultation, clindamycin was reintroduced and flucloxacillin discontinued. The antimicrobial regimen at that point comprised piperacillin/tazobactam, linezolid, rifampicin, caspofungin, and clindamycin. Caspofungin was stopped after 14 days of treatment.

His subsequent ICU course was complicated by peripheral edema, profound weakness, abdominal distension, vomiting, and suspected aspiration pneumonia. NG feeding was halted, and total parenteral nutrition was initiated and gradually increased to full rate. He became delirious, requiring antipsychotic treatment.

By Day 20, infection markers had begun to decline and the patient showed clinical improvement. Linezolid was discontinued, and plans were made to stop piperacillin/tazobactam, clindamycin, and rifampicin in the next 10 days. By Day 24, he began physiotherapy and successfully trialed a T-piece. A speaking valve was introduced on Day 28, and by Day 30, antibiotics were discontinued and he was decannulated, at which point he was tolerating oral intake. Over the following days, he showed steady improvement, mobilizing independently by Day 33, stepping down to the ward on Day 34, and ultimately being discharged home on Day 37.

**Figure 6:**
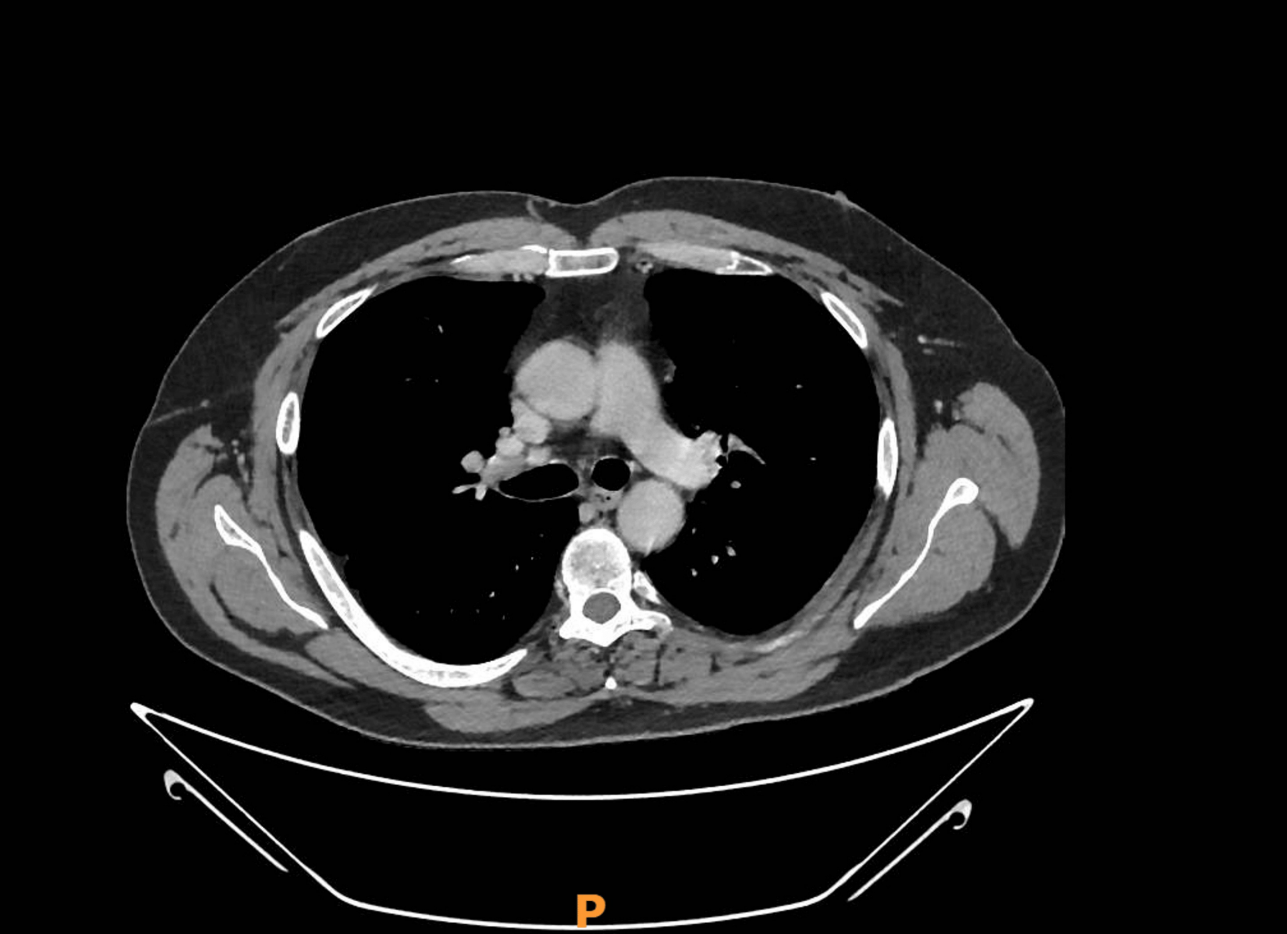
CT scan demonstrating resolution of the previously noted pneumonia in the middle lung zones

At outpatient follow-up, he demonstrated residual restrictive pulmonary changes and diastasis of the rectus abdominis, with no indication for surgical intervention. He was referred to the chronic pain team and advised on lifestyle modifications. CT scan performed has shown complete resolution of the previous pneumonia (Figure 6).

## Discussion

In this case, the presence of a cutaneous lesion, a history of previous S. aureus sepsis, and imaging findings of cavitating lung nodules with central necrosis were key indicators prompting suspicion of PVL-SA. Empirical antibiotic therapy was escalated appropriately, and, following infectious disease consultation, therapy was modified to include linezolid, clindamycin, and rifampicin. This regimen reflects current UK recommendations, which emphasize the use of protein synthesis inhibitors (e.g., clindamycin or linezolid) to reduce toxin production, in addition to agents active against S. aureus [9]. Although the patient was not confirmed microbiologically as PVL-positive until the PCR result, the clinical suspicion was high, and treatment was initiated accordingly.

The 2008 UK Guidance on the Diagnosis and Management of PVL-Associated Staphylococcus aureus Infections, issued by the Health Protection Agency (HPA), provides a framework for the identification and management of PVL-SA [9]. The guidance stresses the importance of early recognition, particularly in patients with recurrent soft tissue infections, pneumonia with cavitation, or close contact outbreaks [9]. It recommends obtaining cultures from all relevant sites and initiating appropriate antibiotic therapy that includes toxin-suppressing agents [9]. Notably, the guidance advises infection control measures, including decolonization for affected individuals and close contacts, especially in cases of recurrent or clustered infections [9].

This case also underscores the value of multidisciplinary input involving microbiology, infectious disease, intensive care, ENT, and respiratory teams. The decision to pursue broad-spectrum antibiotics and antifungal agents initially was reasonable, given the differential diagnosis that included invasive fungal disease and vasculitis. However, the subsequent refinement of treatment based on clinical progression and emerging microbiological data likely contributed to the patient's eventual recovery.

## Conclusions

In conclusion, PVL-SA remains an important differential in patients presenting with rapidly progressive pneumonia, especially when accompanied by soft tissue infection or a history suggestive of prior S. aureus infection. This case demonstrates the hallmark features of PVL-SA infection, with rapid progression from skin and soft tissue involvement to severe necrotizing pneumonia in an otherwise healthy adult. Adherence to national guidance, timely escalation of care, and targeted antimicrobial therapy are crucial to optimizing outcomes in this potentially fatal condition. This case report particularly highlights the benefits of early intervention in conditions that might otherwise be fatal.
